# Simultaneous Cryogenic Radical and Oxidative Coupling Polymerizations to Polyaniline/Polyacrylamide Conductive Cryogels for Gas Sensing

**DOI:** 10.3390/gels8090556

**Published:** 2022-09-01

**Authors:** Xiao-Xiao Guo, Shi-Chang Hou, Hui-Juan Li, Jun Chen, Abdul Haleem, Wei-Dong He

**Affiliations:** 1 Department of Polymer Science and Engineering, University of Science and Technology of China, Hefei 230026, China; 2 School of Pharmacy, China Pharmaceutical University, Nanjing 211198, China

**Keywords:** cryogels, electric conductivity, gas detection, polyaniline, polyacrylamide, simultaneous polymerization

## Abstract

The macro-porous structure of polymer cryogels provides an appropriate channel for the adsorption and transport of substances, endowing its application in the field of electrochemical sensing. The combination mode of a polymer matrix and electro-active substance, particularly the distribution of an electro-active substance in the matrix, has an important effect on the overall performance of the sensor. In this work, through the simultaneous oxidation coupling polymerization of aniline (ANI) and radical polymerization of acrylamide (AAm) under cryogenic condition, conductive composite cryogels were prepared, aiming for the uniform distribution of PANI in the PAAm matrix. The possibility of simultaneous polymerizations was symmetrically investigated, and the obtained PANI/PAAm cryogels were characterized. Due to the acid-doping of PANI, the electrical conductivity of PANI/PAAm cryogels could be modulated with acidic and basic gases. Thus, the performance of the gas sensor was studied by making conductive PANI/PAAm cryogel sheets as resistive sensor electrodes. We found that the content of PANI, the sheet thickness and the dry/wet state of the cryogel influenced the response sensitivity and rate as well as the recovery properties. The response duration for HCl and NH_3_ gas was shorter than 70 and 120 s, respectively. The cyclic detection of HCl gas and the alternate detection of NH_3_/HCl were achieved. This gas sensor with advantages, including simple preparation, low cost and high sensitivity, would have great potential for the application to monitor the leakage of acidic and basic gases.

## 1. Introduction

At present, factory manufacture is one main cause of air pollution due to the presence of injurious gases in the discharging gas and their incident leakage. Although people have taken various processes to avoid these problems, they are not fundamentally solved. A large number of irritating and corrosive gases, particularly acidic and basic gases, which are used and generated in industrial production, cause serious harm to the health of factory workers and endanger the nearby inhabitants.

Hydrochloric acid can corrode human skin and damage the respiratory organs, gastrointestinal organs, eyes and skin [1]. Most discarded hydrochloric acid comes from factories, and some is from the combustion of plastics, such as poly(vinyl chloride) [2]. Ammonia is a highly toxic gas that can burn the skin, eyes and mucous membranes of respiratory organs. Inhaling too much ammonia can cause lung swelling and even death. It is also widely used in chemical fertilizer, pesticides, dyes, explosives, refrigeration, plastics and so on [3,4]. Therefore, it is necessary to prepare a sensor that can constantly monitor the discarding and leakage of those harmful gases.

Metal oxides [5], carbon nano-materials [6] and conductive polymers [7] are commonly used as gas sensors, and are mainly divided into resistive and capacitive sensors. Among them, metal oxide as a sensor has higher sensitivity at high operating temperatures, from 150 to 500 °C [8]. However, a high operating temperature will not only increase the cost and energy consumption but also decrease the sensor stability [9,10]. Carbon nano-materials, due to few functional groups on the surface, have weak chemical adsorption and reactions with gases [10]. In addition, carbon nano-materials exhibit strong π–π interactions and high surface energy and are prone to aggregation and reduced sensitivity to gases [11]. Conductive polymers as gas sensors can not only overcome the shortcomings of metal oxide sensors by working at room temperature [12] but also show attractive sensitivity by modulating their chemical and morphologic structures. Among the conductive polymers, such as polyaniline (PANI) [13], polypyrrole [14,15,16] and porphyrinated polyimide [17], PANI is favored by many researchers because of its simple synthesis, high stability and excellent electrical conductivity, along with the adjustable electrochemical performance through both redox and acid doping [18,19,20,21,22]. Therefore, PANI-based hydrogels are excellent materials as sensors to detect acidic and basic gases.

Since they have poor processability, conductive polymers are usually incorporated in a polymer matrix, such as polymeric hydrogels and membranes, to act as sensors and actuators. Composite hydrogels of conductive polymer and its matrix are commonly prepared through two-step routes with vinyl polymer; for example, (1) oxidative coupling polymerization of ANI, pyrrole or thiophene inside a hydrogel, whose polymer is commonly produced through the radical polymerization of a vinyl monomer; and (2) radical polymerization of vinyl monomer or crosslinkage of polymeric precursor in the presence of a conductive polymer, which is produced through oxidative coupling polymerization. 

Those preparation routes are naturally originated from the fact that both polymerizations are of the radical mechanism, and different radicals from oxidative coupling polymerization inhibit radical polymerization. Through those two-step routes, the uniform distribution of a conductive polymer in a hydrogel matrix has been seriously considered to construct a continuous conductive pathway. Therefore, different strategies have been developed to achieve this goal [18,19,20,21,22]. 

Our group prepared composite hydrogels of polyacrylamide-*g*-polyaniline (PAAm-*g*-PANI) through *γ*-ray copolymerization of acrylamide (AAm) and *N*-(4-aminophenyl)acrylamide (APAM) followed by oxidative coupling polymerization of ANI inside PAAm hydrogels [23]. The presence of aminophenyl groups facilitated PANI grafting onto hydrogel pore-wall to result in the uniform distribution of PANI. Benefitting from the acid-doping of poly(acrylic acid) (PAAc) for PANI and polypyrrole (PPy), our group prepared PAAc-PANI and PAAc-PPy cryogels with well-distributed conductive polymers [24]. Herein, we validate the opportunity to obtain uniform PANI/PAAm cryogels through the concurrence of oxidative coupling polymerization and radical polymerization under cryogenic condition.

Polymeric cryogels refer to the hydrogels or organic gels prepared by the cryo-polymerization of monomers or cryo-gelation of polymeric precursors at a temperature where the solvent is mainly in a crystalline state. The cryogels have an interpenetrated macro-porous structure, quick adsorption to chemicals and a rapid response to external stimuli [25,26,27,28]. Hence, using cryogels as the matrix of a gas sensor can improve the dispersibility of the sensing material, provide more reaction sites, increase the contact area with the gas and further improve the sensitivity of the sensor. Nevertheless, most cryogels are not conductive and cannot be directly used as a sensor. Thus, conductive polymers, such as PANI, should be introduced into one matrix to achieve the functions of composite cryogels. 

Simultaneous cryogenic radical and oxidative coupling polymerizations would facilitate the formation of composite cryogels with uniform distribution of conductive polymer in the matrix. However, as far as we know, there is only one report about the concurrence of oxidative coupling polymerization and radical polymerization in the presence of sulfuric acid (1 M) [29], in which the reason was not disclosed.

In this paper, the composite conductive cryogels of polyaniline/poly(acrylamide-*N,N′*-methylenebisacrylamide) (PANI/PAAm) were successfully synthesized by simultaneous cryo-polymerizations. The conditions and some features of such simultaneous cryo-polymerizations of acrylamide and aniline were investigated. Then, the composite cryogel sheets were prepared as the electrode, and the relationship between the electrode conductivity and the content of doped gas was studied to reveal the possibility to sense different gases.

## 2. Results and Discussion

### 2.1. Simultaneous Polymerizations of AAm and ANI under Cryogenic Condition

It is well known that aniline is one retardant of radical polymerization of vinyl monomers. Therefore, the composites of vinyl polymers and PANI are frequently prepared through physical blend or two-stage polymerization route, radical polymerization of vinyl monomer followed by oxidative coupling polymerization of ANI or versus. 

Eisazadeh and Kavian [29] ever reported the copolymerization of aniline and styrene (St) in emulsion state, investigated the influences of surfactant and found the presence of two monomer residues in the so-called copolymers. However, there was no explanation about no interference of ANI to radical polymerization of St. We assume that the protonation of ANI is the key-point, where the protonated aniline and its inheritors, such as nitrenium cations would have decreased activity to retard radical polymerization of vinyl monomers.

One strong acid (HCl) and two weak acids (H_3_PO_4_ and phytic acid were chosen to disclose the influence of the protonation. Phytic acid, inositol hexaphosphate (C_6_H_6_[OP=O(OH)_2_]_6_), has six phosphoric groups, endowing it the abilities as both the acid dopant and the cross-linker to obtain conductive hydrogels [30,31]. Thus, four mixtures of ANI and acid with the molar ratios shown in Table 1 were prepared in D_2_O to check the protonation of aniline. The ^1^H NMR signals of phenyl protons vary noticeably after the addition of different acids as shown in Figure S1A. 

Without protonation by any acid, ANI shows its two signals of phenyl protons at 6.73 and 7.12 ppm. After the protonation with strong acid HCl, both signals shift to high field location at 7.01 and 7.19 ppm. In the presence of H_3_PO_4_, those two signals become close with each other with chemical shifts of 7.05 and 7.13 ppm, assumed to be caused by the stronger nucleophilicity of the phosphate anion. 

In the PA case, only one broad signal at 6.99 ppm is observed for phenyl protons while the signal from and D_2_O also become broad, suggesting that phytic acid has higher effect on the change of chemical structure. Their FTIR spectra (Figure S1B) demonstrate that the signals of N-H absorbance from aniline without protonation (3100–3500 cm^−1^) turn one broad signal, and the signals of C-H absorbance nearby 3000 cm^−1^ red-shift and widen, which could also support the change of chemical structure with interfering ability of different acids.

With the above ratios of ANI to acid and the molar ratio of ANI to AAm at 1:2.4, the polymerization mixtures were prepared in water, and their simultaneous cryo-polymerizations were performed at −25 °C, while the polymerization of 1/5PA-ANI-AAm-H at 10 °C was also done. First, the results of simultaneous cryo-polymerizations with different acids were compared in appearance. As shown in Figure S2A-1, self-stand columnar cryogels were obtained with PA and HCl (1/5-PA-ANI-AAm and 6/5-HCl-ANI-AAm). 

After being soaked in water for 6 h, their extract liquids were colorless and transparent (the second and fourth vials). After the addition of one thin iodine solution, color remains a little yellow (Figure S2A-2), indicating residual monomer was not detected. However, 6/5-H_3_PO_4_-ANI-AAm resulted in black liquid mixture (Figure S2A, the fifth vial). After high-speed centrifugation, the left solution was yellowish, probably caused by the presence of ANI oligomer. 

Upon the addition of iodine solution, the color turned brownish (the left sixth vial in Figure S2A-2) because I_2_ could act the oxidative dopant for ANI oligomer. The strong acid of HCl facilitated the formation cryogels through simultaneous cryo-polymerizations but weaker acid of H_3_PO_4_ failed. On the contrary, PA, as another weaker acid, produced cryogel at the same molar ratio of phosphoric groups.

Secondly, the amount of PA was decreased to check its influence on protonation degree. As shown in Figure S2B, 1/7-PA-ANI-AAm mixture with less PA addition formed one soft cryogel with easy deformation (the left first vial). The extract after soaking the cryogel in water for 6 h showed turquoise color and became brown with the addition of iodine solution (the left second vial in Figure S2B). Comparison with 1/5-PA-ANI-AAm mixture suggests that excess amounts of PA should be necessary to obtain the self-stand PA-PANI/PAAm cryogel, whose increase of mechanical strength should be attributed to PA crosslinkage.

Thirdly, the temperature of simultaneous polymerization was also examined (−25 °C vs. 10 °C). As shown in Figure S2C, the self-stand hydrogel with lower strength was obtained from 1/5-PA-ANI-AAm-H mixture at 10 °C. The extract after soaking the hydrogel in water was transparent and faint yellow and kept the color of iodine solution after its addition, indicating that soluble ANI oligomer would be hardly produced with sufficient phytic acid. Cryo-gelation has lower critical gelation concentration than common gelation at elevated temperature [32], which offers a reason why the 1/5-PA-ANI/AAm cryogel was stronger than its hydrogel.

**Figure 1:**
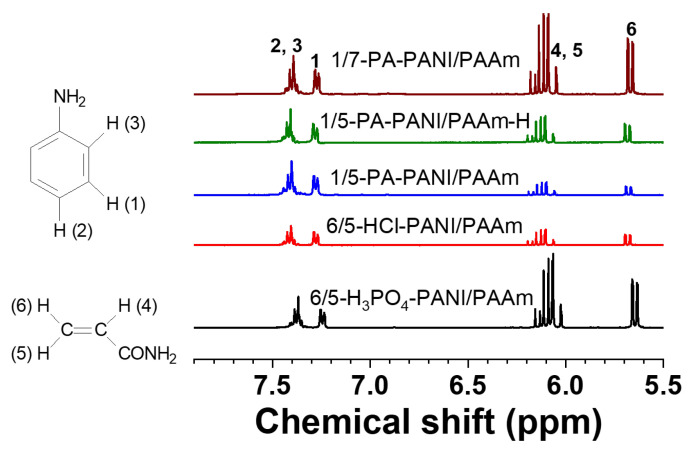
^1^H NMR spectra of the extracts from the five gels with D_2_O.

Quantitative analysis was further executed to evaluate the simultaneous polymerization. First, 5 mg of the above dry gels was soaked in D_2_O for 6 h, and the ^1^H NMR spectra of the liquid extracts were recorded. Shown in Figure 1 are the local spectra, where the signal from D_2_O was used as the inner standard with its integrate height adjusted to the same. The amounts of residual AAm and ANI monomers varies with the condition of simultaneous polymerizations. From the integrate height, the ratio of ANI monomer in the five extracts of 1/7-PA-PANI/PAAm, 1/5-PA-PANI/PAAm-H, 1/5-PA-PANI/PAAm, 6/5-HCl-PANI/PAAm and 6/5-H_3_PO_4_-PANI/PAAm is 2.11:2.50:1.92:1.00:3.02 and that of AAm monomer is 5.34:1.43:1.00:1.10:6.59, indicating that HCl is the best choice of acid for simultaneous cryo-polymerizations.

The monomer conversions of five simultaneous polymerizations were determined with HPLC and GC based on the built calibrations. As shown in Table 2, with sufficient persulfate, ANI was almost completely polymerized but the polymerization of AAm was found to be dependent on the condition. With H_3_PO_4_ and un-excess PA, AAm conversion was fairly low (about 75%).

Based on the above investigations, it can be seen that HCl and excess PA ensure the success of simultaneous polymerizations of AAm and ANI through two interfering mechanisms and the cryogenic condition facilitates the formation of PANI/PAAm cryogels. Considering the utility of obtained cryogels as the sensor to HCl gas, PA-PANI/PAAm cryogels were used for the following sections due to the dual characters of PA.

### 2.2. Structural Analysis and Gas Adsorption of 1/5-PA-PANI/PAAm Cryogels

**Figure 2:**
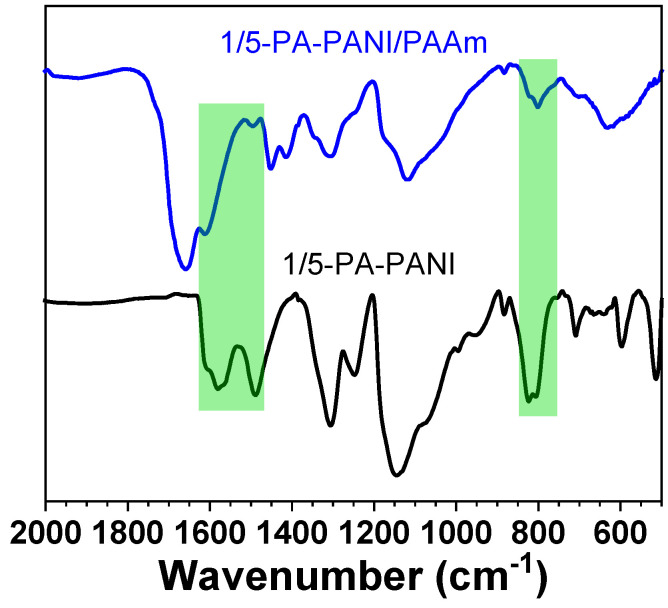
FTIR spectra of PA-PANI and 1/5-PA-PANI/PAAm cryogels.

The chemical composition of 1/5-PA-PANI/PAAm cryogel was confirmed with FTIR spectroscopy with respect to PA-PANI cryogel prepared with PA and ANI at their molar ratio of 1:5. As shown in Figure 2, FTIR spectrum of PA-PANI has two strong absorbance signals at 1579 and 1489 cm^−1^ with similar absorbance intensity, being attributed to the skeleton stretching vibration of phenyl ring with comparable percent of quinonoid and benzenoid structure [33,34]. 

This suggests that the prepared PA-PANI mainly exists in the form of emerald green imine salt rather than the leucoemeraldine (undoped and fully reduced) or permigraniline (doped and fully oxidized) along with the role of PA as an effective dopant [30,31]. PA-PANI also has a strong signal peak near 827 cm^−1^, being assigned to the C-H out-of-plane bending vibration of the di-substituted phenyl ring. As for the FTIR spectrum of PA-PANI/PAAm, the absorbance signals of the skeleton stretching vibration of the phenyl ring vary with the view of wavenumber and intensity, in the presence of PAAm. 

For example, the signal assigned to benzenoid unit of PANI has the wavenumber of 1495 cm^−1^ and becomes weak while that assigned to quinonoid unit likely appears as a shoulder peak. In our previous report, PAAm-*g*-PANI hydrogel was prepared through radical polymerization of AAm followed with oxidative coupling polymerization of ANI with HCl. FTIR spectrum of PAAm-*g*-PANI exhibit those two signals at 1598 and 1528 cm^−1^ with similar absorbance [23]. In the present work, weak absorbance and blue shift suggest the interaction between PANI and PAAm. The signal of phenyl out-of-plane bending vibration also exists. The absorbance band of the C=O stretching vibration peak is observed at 1655 cm^−1^ for AAm units.

**Figure 3:**
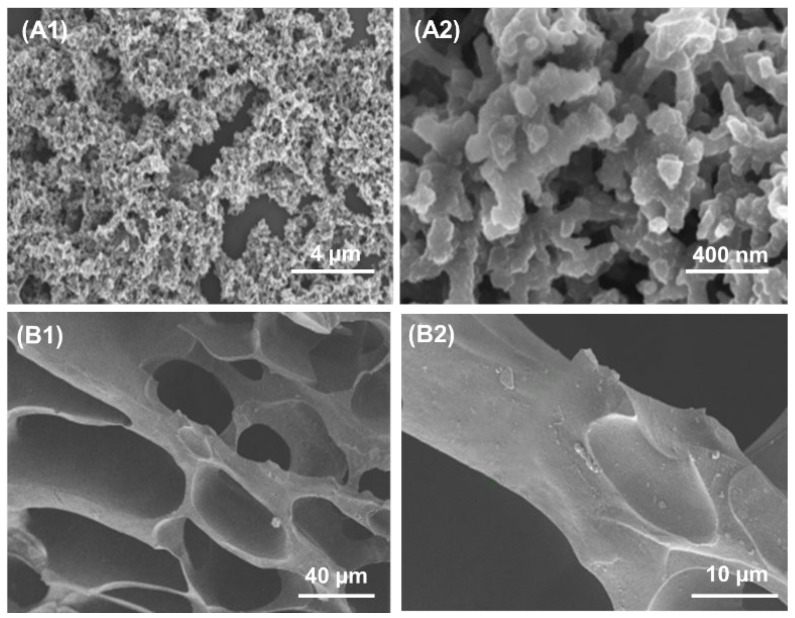
SEM images of PA-PANI (**A1**,**A2**) and 1/5-PA-PANI/PAAm cryogels (**B1**,**B2**).

SEM was used to observe the cryogel morphology. With PA as the dopant and physical cross linker, bulky PANI cryogel was obtained. As shown in Figure 3A, PANI cryogel show porous morphology with PANI particles being interconnected to form the coral-like structure cluster. The three-dimensional porous morphology is beneficial to the continuous channel for electrical conduct. The cryogel produced under cryogenic condition often has interpenetrating macropores with the pore size in the micron range. 

Figure 3B are SEM images of 1/5-PA-PANI/PAAm cryogel with two magnifications. The cryogel shows the honeycomb three-dimensional porous structure and the pore size is large in 50–150 μm. The formation of such morphology is the intrinsic feature of cryogelation with ice crystals as the porogen. Some flakes and small particulates are observed on the cryogel pore-wall, suggesting the possible phase separation between PANI and PAAm in the unfrozen liquid microphase during the simultaneous cryo-polymerizations.

**Figure 4:**
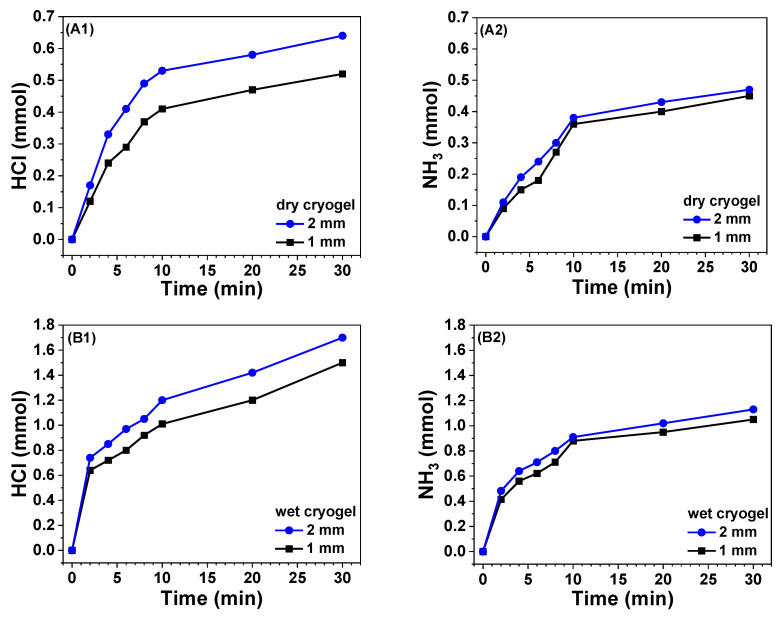
Variations of gas amount adsorbed by dried (**A1**,**A2**) and wet (**B1**,**B2**) sheets of 1/5-PA-PANI/PAAm cryogel.

Gas adsorption is the premise for the obtained cryogel to sense the acidic and basic gases. Thus, 1/5-PA-PANI/PAAm cryogel sheets with the same surface area but two thicknesses of 1 and 2 mm were used to adsorb HCl and NH_3_ gas at 12 ± 1 °C. The as-prepared wet and lyophilized cryogels were compared. The amount of adsorbed gas by the cryogels was determined with titration. The variations of gas amount within 30 min are shown in Figure 4. 

When dry cryogel is encountered, the increase of adsorbed amounts is sharp in 10 min for of both HCl and NH_3_ gas and then slows down. At the same interval, the adsorbed amount of HCl is larger than that of NH_3_, even if there exists PA in the cryogel sheets. As expected, the thicker cryogel sheet adsorbs more both gases than the thinner sheet. When wet cryogel is used, the above trends are also observed. Moreover, due to the presence of water, the wet sheets have a significantly higher amount of adsorbed gases than the dry sheet. After 10 min, HCl amount keeps clear increasing.

### 2.3. Dependence of Cryogel Conductivity on the Adsorbed Amount of HCl and NH_3_

The conductivity of PANI is related to the content of doped acid [30,31,32,35,36,37], the oxidation state of PANI (oxidative degree of PANI) [38] and temperature. In this work, the dependence of cryogel conductivity on the adsorbed gases was investigated from the following aspects: (1) PA/ANI molar ratio (1:5, 1:4 and 1:3); (2) ANI amount (2.5, 3 and 4 mmol); (3) oxidative degree of PANI controlled by ANI/APS molar ratio (4:1, 3:1, 2:1, 1:1 and 2:3; (4) thickness of cryogel sheet (1 and 2 mm); and (5) water content of cryogel sheet (dry and wet), where the dry sheet is obtained by lyophilization while wet sheet is fully swollen and free from surface water. The synthesis information of the relevant cryogel sheets are summarized in Table 3, where the cryogels are named x-y with x and y referring the ratio of PA:ANI and ANI:AAm, respectively.

**Figure 5:**
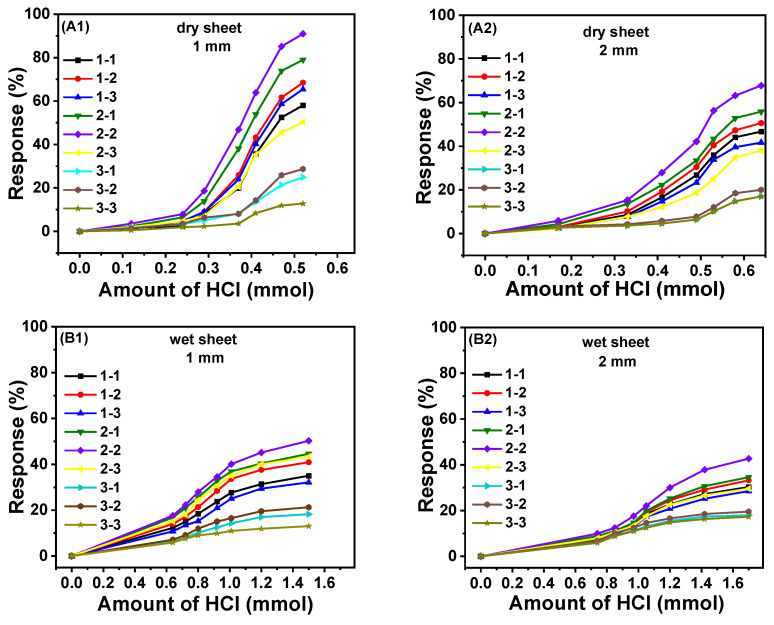
The response of dry and wet cryogel sheets with two thickness ((**A1**): dry, 1 mm, (**A2**): dry, 2 mm; (**B1**): wet, 1 mm, (**B2**): wet, 2 mm) to adsorbed HCl gas.

First, the conductivity changes of nine sheets to HCl gas in dry and wet states are compared within 30 min as shown in Figure 5. Among all the cryogel sheets, the change of conductivity of 2-2 cryogel is the most obvious. According to four figures, the response of 3-y cryogel sheets is generally the lowest compared with other groups with lower PA content. In the composite cryogel, PA acts as not only the acid dopant to PANI but also the physical cross-linker. 

Although the increase of PA amount could increase the electrical conductivity due to the increased protonation degree of ANI benzenoid units, the accompanying increase of crosslinking degree would inhibit the transport of adsorbed HCl gas since higher crosslinking degree leads to less and smaller gaps among polymer chains in the cryogel wall for the diffusion of HCl into the wall interior. In each cryogel group with the same amount of PA, it is found that the sheet with 3 mmol ANI (the middle value) has the highest conductivity in response to adsorbed HCl. 

Regarding the thickness of cryogel sheet, the response of thin sheets (1 mm) was better since its doping state is more effectively changed by adsorbed HCl. Moreover, the conductivity change of dry sheets is clearly higher than that of wet sheets since the dry cryogel sheet with high porosity is more convenient to combine with HCl to achieve higher acid-doping degree. However, as for the wet cryogel sheets, the adsorbed gas should dissolve in water and diffuse through water phase before it combines with PANI component.

**Figure 6:**
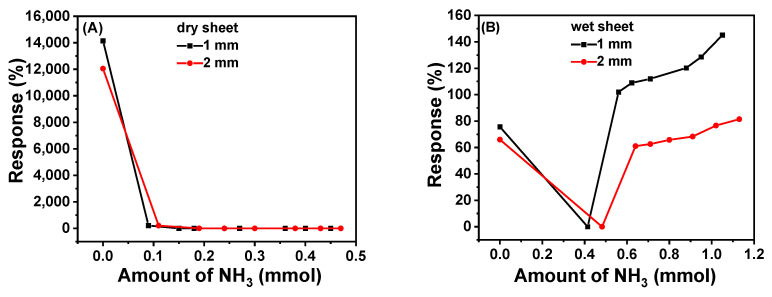
Response of 2-2 cryogel sheets to adsorbed NH_3_ gas ((**A**): dry state; (**B**): wet state).

The response of 2-2 cryogel sheet, i.e., 1/4-PA-PANI/PAAm with ANI:AAm at 1:2 in mole, to NH_3_ gas is shown in Figure 6. It can be seen that the de-doping effect of PANI is more obvious than the doping effect with HCl when dry sheets are used, considering the sharp decrease of response with low gas content. At about 0.1 mmol of NH_3_ content, the conductivity of both dry sheets with 1 or 2 mm thickness becomes almost zero. 

On the contrary, wet cryogel sheets exhibit different response behavior compared with dry sheets. Their response, calculated based on the lowest current upon adsorbing NH_3_, is much lower than that of dry sheets. Up to 0.4–0.5 mmol of adsorbed NH_3_, the conductivity drops nearly to zero, due to the de-doping. With much more adsorbed NH_3_, the conductivity keeps increasing because of the partial ionization of the formed ammonia water (NH_3_·H_2_O), ${\mathrm{NH}}_{3}\cdot H_{2}O\;⇌{\;\mathrm{NH}}_{4}^{+}+{\mathrm{HO}}^{-}$.

It is known that the electrical conductivity of PANI is also related to the oxidation degree of PANI besides acid-doping. Therefore, cryogel sheets with other molar ratios of ANI to APS were also evaluated along with 2-2 cryogel at the ratio of 4:1. As shown in Figure S3, when the ratio of ANI to APS is 4:1, 3:1 and 2:1, the conductivity of three dry cryogels shows the same change behavior in response to HCl and NH_3_ gases. In those cases, PANI is in the intermediate oxidation state with the coexistence of quinonoid and benzenoid units. 

PANI in those composite cryogels has a higher molar fraction of benzenoid than quinonoid, which favors the acid-doping to exhibit higher ability to sense acidic and basic gases. It is well known that a slight excess of oxidant compared to ANI is commonly adopted to reach the similar molar fraction of quinonoid and benzenoid for high conductivity and the best yield of PANI [39]. In the present cases, when the oxidant amount is equal or greater than ANI amount, the content of benzenoid units in PANI significantly increases, lessening the acid-doping effect.

### 2.4. Sensing Performance of PA-PANI/PAAm Cryogel to HCl and NH_3_

**Figure 7:**
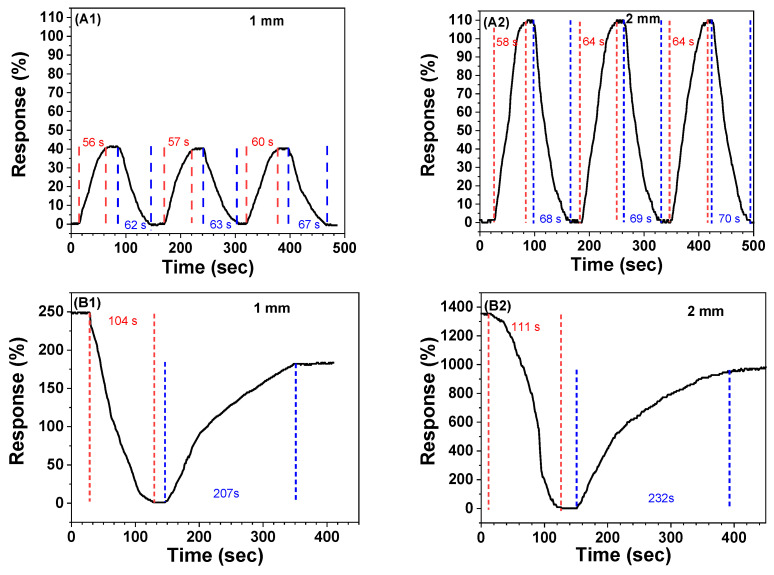
(**A**) Cyclic response of dry 2-2 cryogel with thickness of 1 mm (**A1**) and 2 mm (**A2**) to HCl gas. (**B**) Response of dry 2-2 cryogel with thickness of 1 mm (**B1**) and 2 mm (**B2**) to NH_3_ gas.

Based on the results in Figure 5, 2-2 cryogel sheets, 1/4-PA-PANI/PAAm at ANI:AAm of 1:2 in mole, were used to assess the sensing performance. Shown in Figure 7 are the response variations during three cycles to pump HCl gas and air in turn across the cryogel sheets with two thickness values. Upon pumping HCl gas from 36 wt% solution, the response increases within about 60 s to steady values of 40% and 108% for the cryogel sheets with a thickness of 1 and 2 mm, respectively. 

Wang et al. prepared quartz crystal microbalnace (QCM) sensors coated with PANI-funtionalized polycaprolactam naofiber-net-binary structured membranes to detect trace HCl gas based on QCM resonance frequency change. They found that QCM frequency dropped to constant values within about 50 s [40]. Although detection limit was as low as 7 ppb, the QCM instrument with high price should be used. The thicker sheet has a little longer response time. After pumping air into the test chamber, adsorbed HCl gas is blown off gradually and the response correspondingly decreases to zero within 60–70 s for both cryogel sheets. As discussed above, the thicker sheet has more PANI amount and longer diffusion pathway of gas, causing longer duration to reach the steady adsorption and larger amount of adsorbed gas. It is noticeable that the response duration has the increase tendency with the cycle number.

As shown in Figure 7B, when NH_3_ gas is pumped from the volatilization of 25 wt% NH_3_·H_2_O solution, the circuit current decreases to one bottom value in 104 and 111 s for two dry cryogel sheets with two thickness values. Kukla et al. [41] proposed an ammonia sensor of emeraldine PANI film with HClO_4_ dopant and found the time needed to reach a stable electrical resistance was not more than 2 min, which is comparable with our result. However, being contrary to HCl case, when air is in turn pumped insides, the circuit current does not restore the original value. The electrical conductivity losses are 81% and 72%. 

The restoring durations are 207 and 232 s for the cryogel sheets of 1 and 2 mm, respectively. The longer recovery duration should be caused the strong interaction between basic gas and the acid of PA in the cryogel sheet. The incomplete recovery suggests that ammonium salt should be formed. If the cryogel sheet is exposed in NH_3_ gas for 1 h, the accumulation of formed ammonium salt will cause hardly the conductivity recovery [41]. Thus, the cycle response by alternate charging NH_3_ and air has not been tested. Compared with Figure 7A1,A2, it can be seen that increasing cryogel sheet thickness can enhance the response but the response duration enlarges.

**Figure 8:**
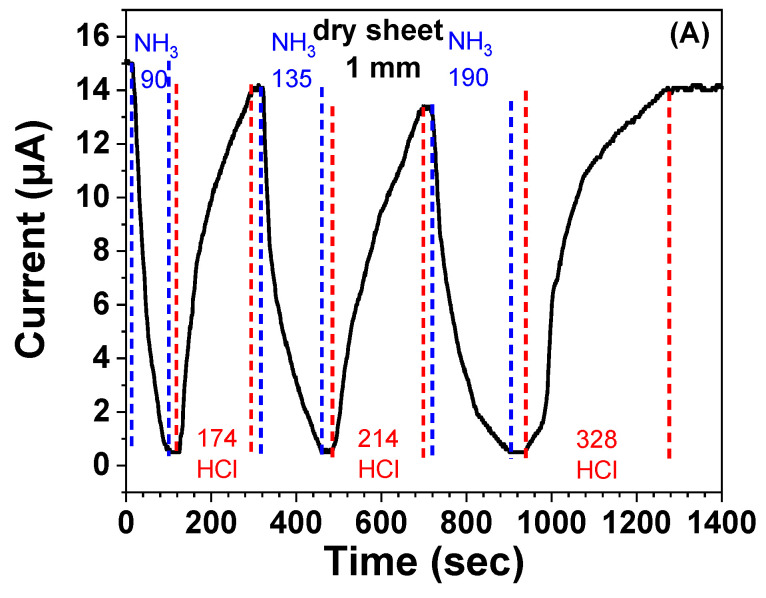
Cyclic response of dry 2-2 cryogel with a thickness of 1 mm to NH_3_ and HCl gas.

The cyclic response evaluation with alternate charging NH_3_ and HCl gas has also been performed with 2-2 dry cryogel sheet at 1 mm thickness. The three-cycle result is shown in Figure 8, where the circuit current is used directly to demonstrate the conductivity change. When the circuit current drops to about 0.5 μA with charging NH_3_ gas, the analysis gas is alternately changed with HCl gas until the increased current does not change within 20 s. 

It can be seen that the alternate introduction of HCl gas would recover the conductivity to the approximate level of the original value if the HCl charging is sufficiently long, which is confirmed in the third cycle. Although the response duration tends to increase with cycle number, the initial changes in each cycle is sharp, suggesting the possibility of the obtained PA-PANI/PAAm cryogel to detect alternately the leakage of NH_3_ and HCl gas.

## 3. Conclusions

In this work, the oxidative coupling cryo-polymerization of aniline and radical cryo-polymerization of acrylamide were simultaneously achieved, and PANI/PAAm composite cryogels with macro-porosity and uniform distribution of PANI were successfully obtained to effectively detect the gases of HCl and NH_3_. Strong acids, such as HCl, and one weak acid of phytic acid were found to guarantee the sufficient protonation of ANI to depress its retardant effects in the radical polymerization of acrylamide and ensure almost complete conversion of acrylamide apart from ANI. 

Interestingly, the results about the adsorption of two gases revealed that PA-PANI/PAAm cryogel prefers HCl gas in spite of the presence of PA in the cryogel. With PA-PANI/PAAm cryogel sheets, the dependences of electrical conductivity on different synthesis parameters were symmetrically investigated, and the optimal condition was confirmed. The optimal cryogel sheets of PA-PANI/PAAm were used to evaluate the sensing performance with regards to the response sensitivity and recovery possibility. Within three cycles of alternate charging HCl gas and air, the response duration and recovery duration were found to be no more than 65 and 70 s, respectively. 

Due to the formation of ammonium salt, the electrical conductivity failed in restoring to the original value upon alternate pumping NH_3_ gas and air. The thicker sheet had higher response sensitivity but a slightly longer duration. However, during three cycles of alternating atmospheres of NH_3_ and HCl, repeatable detection for both gases was achieved although the duration became longer with the cycles. Therefore, the PA-PANI/PAAm cryogel obtained from simultaneous cryo-polymerizations is a potential candidate as a sensor to monitor the leakage of acidic and basic gases.

## 4. Experimental

### 4.1. Materials

Aniline (ANI, CR, Sinopharm Chemical, Shanghai, China) was used after distillation. Ammonium persulfate (APS, AR, Sinopharm Chemical) and potassium persulfate (KPS, AR, Sinopharm Chemical) were used after recrystallization with water. Hydrochloric acid (HCl, 36 wt%, AR, Sinopharm Chemical), phosphoric acid (H_3_PO_4_, 85 wt%, AR, Sinopharm Chemical), ammonia (NH_3_·H_2_O, 25 wt%, AR, Sinopharm Chemical), L-ascorbic acid (VC, AR, 3A Chemical, Shanghai, China), phytic acid solution (PA, 70 wt% in H_2_O, Aladdin, Shanghai, China), methanol (AR, Sinopharm Chemical), methylene dichloride (AR, Sinopharm Chemical), ethyl acetate (HPLC, Macklin, Shanghai, China), acrylamide (AAm, 99.0%, Aladdin) and *N,N′*-methylenebisacrylamide (MBA, 99%, Aladdin) were used as received. Deionized water was used in all experiments.

### 4.2. Investigation of Simultaneous Polymerization of Aniline and Acrylamide

#### 4.2.1. Structural Change of Aniline Protonated with Different Acids

To ensure the parallel protonation of aniline (5.0 mmol), the amount of acid was chosen to be 6.0 mmol for HCl and H_3_PO_4_, 1.0 mmol for PA, respectively. The acid was dissolved in D_2_O (5 mL) individually. Then, ANI (5.0 mmol) was added, and the structural change caused by the protonation of aniline was characterized by FTIR and ^1^H NMR.

#### 4.2.2. Study of Simultaneous Cryo-Polymerizations in the Presence of Different Acids

ANI (5 mmol) and one acid (6.0 mmol for HCl and H_3_PO_4_, 1.0 mmol for PA) were added into the centrifuge tube and dispersed in water (1 mL) to obtain Dispersion A. Dispersion B was obtained by adding AAm (12 mmol) and MBA (0.15 mmol) into another centrifuge tube and dispersed in water (2 mL). After Dispersion B was pre-frozen at −25 °C for 10 min and kept in an ice bath for thawing to avoid possible reactions, the aqueous solutions of VC (0.5 mL, 0.07 mmol) and KPS (0.5 mL, 0.06 mmol) were added in turn under ice bath conditions. 

After Dispersion A was pre-frozen at −25 °C for 7 min and kept in an ice bath for thawing, APS solution (1.0 mL, 1.67 mmol) was added. Finally, the two dispersions were mixed by vigorous shaking and was placed at −25 °C for 48 h. Then, the sample was taken out and thawed. After being immersed in water (50 mL) for 6 h, the soaking solution was divided into three parts to detect the unpolymerized monomers of ANI and AAm in qualitative and quantitative ways, respectively. For the qualitative detection of residual AAm, iodine solution (0.02 mol·L^−1^) was added to the soaking solution.

For the quantitative detection of residual AAm, the soaking solution was brominated, extracted with ethyl acetate, and then the AAm content was determined by gas chromatography. For the quantitative detection of residual ANI, the soaking solution was extracted by dichloromethane, and then the aniline content was determined by high-pressure liquid chromatography. A small piece of as-prepared sample was directly lyophilized, and the dried sample (5 mg) was immersed in D_2_O (1 mL) for ^1^H NMR analysis of residual monomers.

### 4.3. Synthesis of PANI/PAAm Cryogels through Simultaneous Cryo-Polymerizations

First, the cryo-polymerization of ANI in the presence of phytic acid was performed to prepare PANI doped and cross-linked with phytic acid (PA-PANI). PA solution (1.25 mmol) and APS (1.67 mmol) were added into a 10 mL centrifuge tube and dissolved in water (5 mL). Kept in an ice-bath, ANI (5.0 mmol) was added, and the centrifuge tube was shaken vigorously. Then, the mixture was placed at −25 °C for 48 h. Dry PA-PANI cryogel was obtained by lyophilization.

Then, different PANI/PAAm cryogels were synthesized with the variations of ANI and PA amounts. One typical procedure is given as follows. ANI (5 mmol) and PA solution (1.25 mmol) were dispersed in water (1.0 mL) in a centrifuge tube (Dispersion A). AAm (12 mmol) and MBA (0.15 mmol) were dispersed in water (2.0 mL) in another centrifuge tube (Dispersion B). Both dispersions were kept at −25 °C for pre-freezing and then in an ice bath for thawing. APS solution (1.67 mmol, 1 mL) was added to Dispersion A, while VC solution (0.07 mmol, 0.5 mL) and KPS solution (0.06 mmol, 0.5 mL) were added in turn into Dispersion B. 

Immediately, the solutions in both tubes were mixed well by vigorous shaking. The polymerization was performed at −25 °C for 48 h. Afterwards, the obtained cryogel was took out, thawed and soaked in water for 6 h to remove unreacted monomers and un-crosslinked polymer. During the soaking, the water was changed every 2 h. Dried PA-PANI/PAAm conductive cryogels were obtained by lyophilization.

### 4.4. Fabrication of Gas Sensor

Before the fabrication of the gas sensor, the mold for the sensor sheet was prepared. First, a silicone rubber plate (0.1 and 0.2 cm in thickness) was cut into a hollow rectangle at 5.5 cm × 1.0 cm. The button side of the mold was fixed on one glass slide with super glue. The upper side was coated with vacuum grease and covered with another glass slide. The preparation of sheet pre-solution was the same as Section 4.3. After obtaining the pre-solution, the mold was pre-cooled with liquid nitrogen for 2 min. Then, the pre-solution was dripped into the mold to fill the mold completely. 

**Figure 9:**
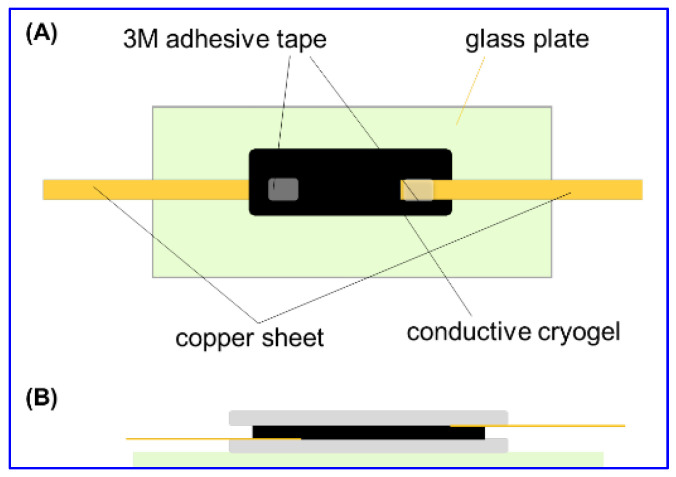
Fabrication of electrode for gas sensor with the overhead view (**A**) and cross-sectional view (**B**) of a cryogel electrode.

The mold was sealed by covering the upper glass slide and clamping two glass slides. After the mold was kept −25 °C for 48 h, the cryogel sheet was thawed, soaked in water and lyophilized. To fabricate the gas sensor, two copper sheets were fixed onto one cryogel sheet by contacting both surfaces and ends with 3M double-sided adhesive tape. Then, the electrode was kept between two glass plates as shown in Figure 9. For gas detection, the upper glass plate was removed.

### 4.5. Characterizations and Intruments

Fourier transform infrared (FTIR) spectra were recorded on Nicolet 6700 instrument (Thermo Fisher, Waltham, MA, USA) with the wavenumber range of 4000–500 cm^−1^, scanning step of 4 cm^−1^ and 16 scanning times. For the test samples, ANI protonated with different acids was coated on KBr flakes, while the cryogel was crashed with KBr and pressed into pellets. Proton nuclear magnetic resonance (^1^H NMR) spectra were recorded on Avance 300 instrument (Bruker Biospin, Fällanden, Switzerland) with D_2_O as the solvent. 

Gas chromatography (GC) and high pressure liquid chromatography (HPLC) measurements was performed on GC 6890 (Agilent, Santa Clara, CA, USA) and HPLC 1260 (Agilent, USA), respectively. The porous morphology of cryogels was observed under scanning electron microscope (SEM, Evo18, Carl Zeiss, Oberkochen, Germany) with freeze-dried cryogels sputtered with gold. The electrochemical properties was determined on one electrochemical workstation (CHI760E, Shanghai Chenhua, Shanghai, China).

### 4.6. Study of the Sensing Performance to Different Gases

**Figure 10:**
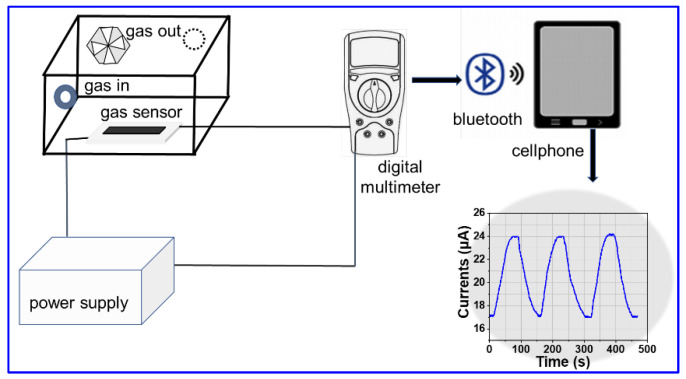
Measurement system for testing gas sensors.

The electrical sensing characteristics of the sensors were measured using a bench system at 10–15 °C, as presented in Figure 10. The system was assembled with the integrated sensor, a OWON digital multimeter [B35T+, Bost, Owon, Zhangzhou, China] connected to cellphone via Bluetooth and a fixed 8 V power supply (UTP3313TFL-Ⅱ, Uni-T, Dongguan, China). The experiment was performed by measuring the circuit current in different gases with various amounts. HCl and NH_3_ gases were obtained through volatilizing from the 36 wt% hydrochloric acid solution and 25 wt% ammonia solution and pumped with nitrogen flow into the test chamber (22.5 L in volume). The gas inside the chamber was uniformly distributed using a fan. All experiments were performed at room temperature, which was about 10–15 °C, and the humidity was about 26 ± 2 RH%.

Through the measurement of circuit current with the digital multimeter, the response of the electrode to acidic gas (HCl) was calculated according to Equation (1).

(1) $$Res=\frac{\left(I_{\mathrm{gas}}-I_{0}\right)}{I_{0}}\times 100\%$$

where *I*_gas_ and *I*_0_ is the circuit current after and before being fed with analysis gas, respectively. The response to basic gas (NH_3_) was calculated according to Equation (2).

(2) $$Res=\frac{I_{\mathrm{gas}}}{I_{\mathrm{gas},\mathrm{steady}}}\times 100\%$$

where *I*_gas,steady_ is the lowest current upon adsorbing sufficient NH_3_ gas.

Since circuit current changed due to doping with adsorbed HCl gas and de-doping with adsorbed NH_3_ gas, their adsorbed amounts were determined with other sheets of different cryogels. Under the same condition as above, the sheets, after being kept in the gas chamber for the same durations as those in sensing evaluation, were taken out and immersed in water for at least 6 h. Then, the content of HCl or NH_3_ in water was determined by the titration with standard solution of NaOH or HCl.

## Figures and Tables

**Table 1:** Simultaneous polymerizations of AAm and ANI with different acids ^a^.

Mixture	Temperature (°C)	Acid	Acid: ANI in Mole	Gel
1/7-PA-ANI-AAm	−25	PA	1:7	1/7-PA-PANI/PAAm
1/5-PA-ANI-AAm-H	10	PA	1:5	1/5-PA-PANI/PAAm-H
1/5-PA-ANI-AAm	−25	PA	1:5	1/5-PA-PANI/PAAm
6/5-HCl-ANI-AAm	−25	HCl	6:5	6/5-HCl-PANI/PAAm
6/5-H_3_PO_4_-ANI-AAm	−25	H_3_PO_4_	6:5	6/5-H_3_PO_4_-PANI/PAAm

^a^ The molar ratio of ANI to AAm is kept as 1:2.4 for all.

**Table 2:** Monomer conversions of AAm determined by GS and ANI by HPLC.

Gel	AAm		ANI	
	Surplus	Conversion	Surplus	Conversion
1/7-PA-PANI/PAAm	103.3 mg	75.8%	3.9	98.3%
1/5-PA-PANI/PAAm-H	16.4 mg	96.2%	3.9	98.3%
1/5-PA-PANI/PAAm	12.5 mg	97.1%	3.3	98.6%
6/5-HCl-PANI/PAAm	14.1 mg	96.7%	3.6	98.5%
6/5-H_3_PO_4_-PANI/PAAm	109.5 mg	74.3%	3.7	98.4%

**Table 3:** The preparation receipt of different cryogel sheets ^a^.

Cryogel	Molar Ratio		Charged Amount		
	PA:ANI	ANI:AAm	ANI	PA	APS
1-1	1:5	1:2.4	2.5 mmol	0.50 mmol	0.63 mmol
1-2		1:2	3.0 mmol	0.60 mmol	0.76 mmol
1-3		2:3	4.0 mmol	0.80 mmol	1.000 mol
2-1	1:4	1:2.4	2.5 mmol	0.63 mmol	0.63 mmol
2-2		1:2	3.0 mmol	0.75 mmol	0.76 mmol
2-3		2:3	4.0 mmol	1.00 mmol	1.00 mmol
3-1	1:3	1:2.4	2.5 mmol	0.83 mmol	0.63 mmol
3-2		1:2	3.0 mmol	1.00 mmol	0.76 mmol
3-3		2:3	4.0 mmol	1.33 mmol	1.00 mmol

^a^ AAm (6 mmol), MBA (0.075 mmol), KPS (0.03 mmol) and VC (0.035 mmol) were added. The total amount of water was 2.0 mL.

## Data Availability

Not applicable.

## Supplementary Materials

The following supporting information can be downloaded at: https://www.mdpi.com/article/10.3390/gels8090556/s1, Figure S1: 1H-NMR (A) and FTIR spectra (B) of ANI protonated with different acids; Figure S2: Digital photos of thawed as-prepared products and the extract before and after the addition of iodine solution; Figure S3: Gas-dependent conductivity changes of 1 mm dry PAMA cryogels prepared with different molar ratios of ANI to APS.
