# Supplementary material for: Simultaneous Cryogenic Radical and Oxidative Coupling Polymerizations to Polyaniline/Polyacrylamide Conductive Cryogels for Gas Sensing
Source: Gels. 2022 Sep 1;8(9):556. doi: 10.3390/gels8090556 (PMC9498737; doi:10.3390/gels8090556)
Supplement: Supplementary file 1 [file gels-08-00556-s001.zip › gels-1844799-supplementary.pdf]

Supporting Information

Simultaneous cryogenic radical and oxidative coupling polymerizations to polyaniline/polyacrylamide conductive cryogels for gas sensing

Xiao-Xiao Guo[1], Shi-Chang Hou[1], Hui-Juan Li[2], Jun Chen[1], Abdul Haleem[1] and Wei-Dong He[1,*]

[1] Department of Polymer Science and Engineering, University of Science and Technology of China, Hefei, Anhui 230026, China.
[2] School of Pharmacy, China Pharmaceutical University, Nanjing, Jiangsu 211198, China. ahlhj@sina.cn
* Correspondence: wdhe@ustc.edu.cn. The first two authors contribute equally.

Protonation of aniline with different acid.

We assume that the structural variations of aniline and its intermediate products due to the protonation would weaken the retardant ability to radical polymerization of vinyl monomers. Thus, the mixtures of aniline with different acids were characterized with ^{1}H-NMR and FTIR spectra, as shown in Figure S1.

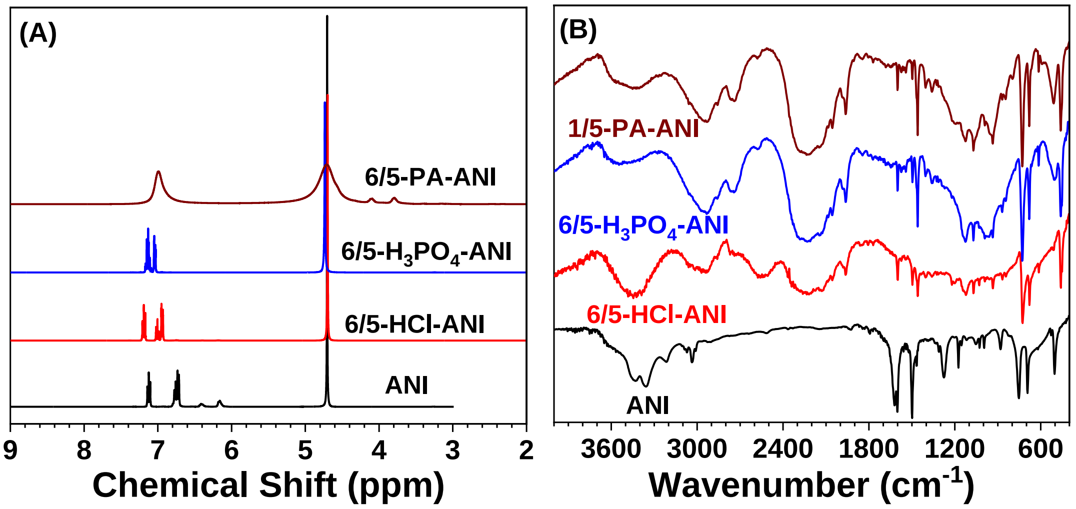

Figure S1. ^{1}H-NMR (A) and FTIR spectra (B) of ANI protonated with different acids.

Appearance observation and qualitative analysis of simultaneous polymerizations

As shown in Table 1, different simultaneous polymerizations were carried out at -25 and 10 °C. The vials with black gels or liquid are referred to the as-prepared products after being thawed, while the identical polymerization condition is annotated to the relevant vial. In each x-1 photo, the vial right next to the vial above mentioned is referred to the extract with soaking the gel in water for 6 h or that after centrifugation (the right first vial in Photo A-1). In each x-2 photo, the vial right next to the vial containing black gel or liguid is referred to the above extract after the addition of thin iodine solution.

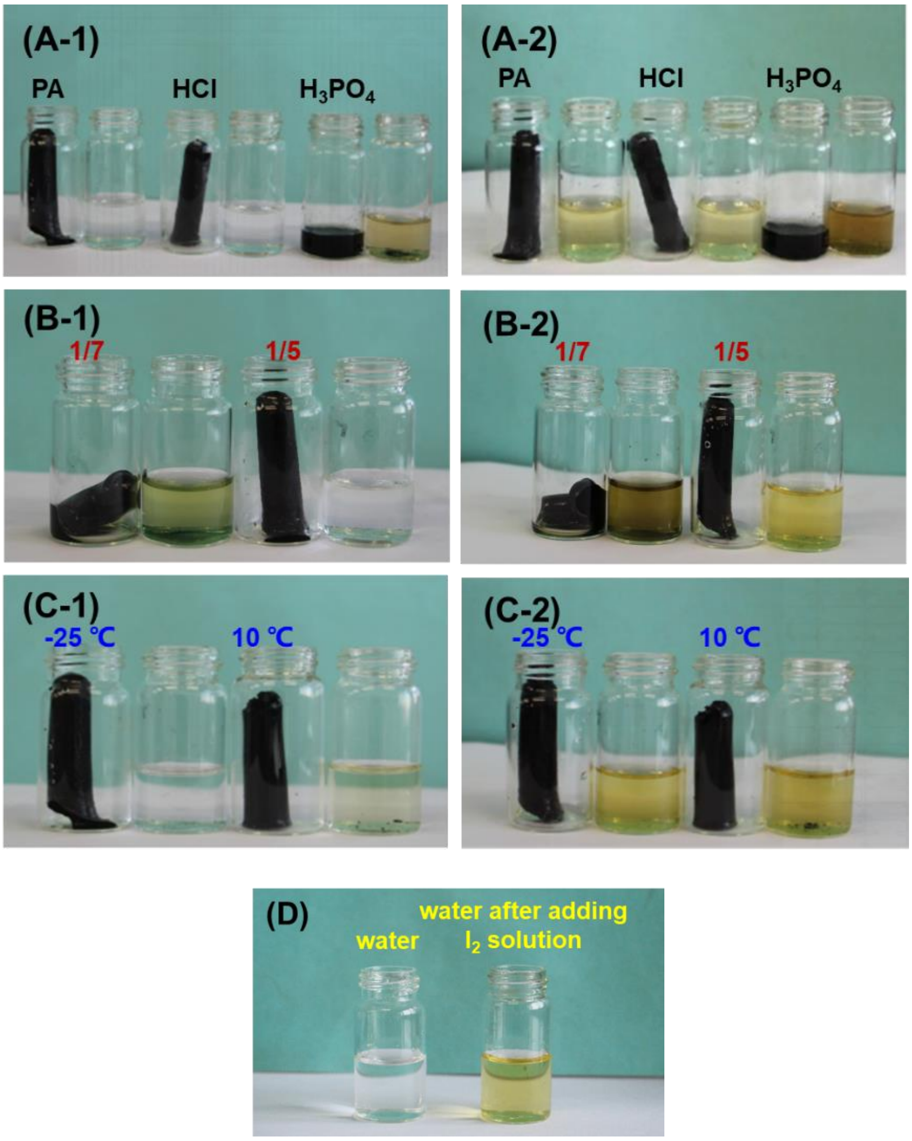

Figure S2. Digital photos of thawed as-prepared products and the extract before and after the addition of iodine solution (A: the products from different acids; B: the products from different PA amounts; C: the products from the same PA amount at different temperatures; D: pure water before and after adding iodine solution).

Influence of APS oxidant on the gas detection of the obtained cryogel sheets from simultaneous cryo-polymerizations

To investigate the influence of APS oxidant on the gas detection of the obtained cryogel sheets from simultaneous cryo-polymerizations, more cryogel sheets with other molar ratios of ANI to APS along with 2-2 cryogel at this ratio of 4 : 1 were synthesized. Their responses to HCl and NH3 are shown in Figure S3.

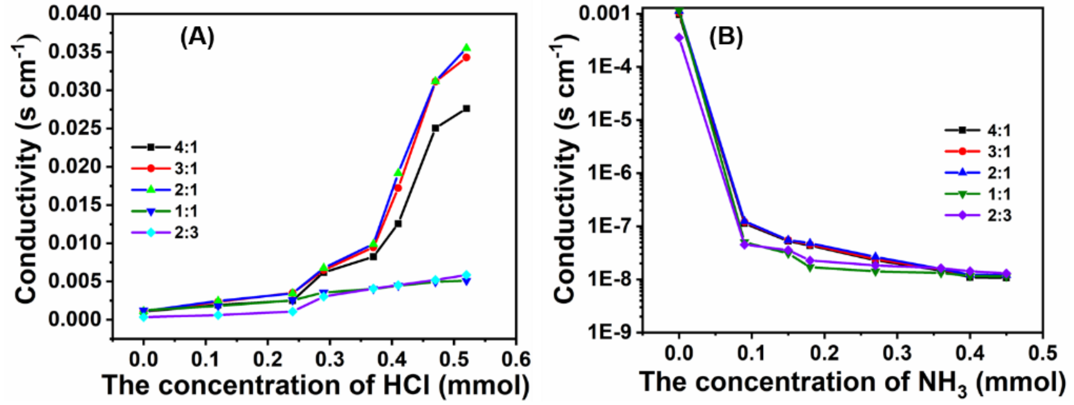

Figure S3. Gas-dependent conductivity changes of 1 mm dry PAMA cryogels prepared with different molar ratios of ANI to APS (where ANI: APS = 4:1, 3:1, 2:1, 1:1 and 2:3) (A: HCl, B: NH_3).
